# Peripheral T-Cell Lymphoma, Not Otherwise Specified: Clinical Manifestations, Diagnosis, and Future Treatment

**DOI:** 10.3390/cancers13184535

**Published:** 2021-09-09

**Authors:** Stefano A. Pileri, Valentina Tabanelli, Stefano Fiori, Angelica Calleri, Federica Melle, Giovanna Motta, Daniele Lorenzini, Corrado Tarella, Enrico Derenzini

**Affiliations:** 1 Division of Haematopathology, Haematology Programme, IEO European Institute of Oncology IRCCS, Via Ripamonti 435, 20121 Milan, Italy; valentina.tabanelli@ieo.it (V.T.); stefano.fiori@ieo.it (S.F.); angelica.calleri@ieo.it (A.C.); federica.melle@ieo.it (F.M.); giovanna.motta@ieo.it (G.M.); daniele.lorenzini@ieo.it (D.L.); 2 Division of Haemato-Oncology, Haematology Programme, IEO European Institute of Oncology IRCCS, Via Ripamonti 435, 20121 Milan, Italy; corrado.tarella@ieo.it (C.T.); enrico.derenzini@ieo.it (E.D.); 3 Department of Health Sciences, University of Milan, Via di Rudinì 8, 20146 Milan, Italy

**Keywords:** peripheral T-cell lymphoma, not otherwise specified, cell morphology, phenotype, gene expression profiling, next-generation sequencing, classification, diagnosis, prognosis, therapy

## Abstract

**Simple Summary:**

The intent of this paper is to critically revise the epidemiology, etiology, main clinical features, histopathology, immunophenotype, molecular characteristics, prognosis, and therapeutic options of peripheral T-cell lymphoma, not otherwise specified (PTCL_NOS). PTCL_NOS represents the commonest category of PTCL. Regarded as an exclusion diagnosis, it has a quite distinctive profile, based on the authors’ experience and data from the literature. New therapeutic agents are discussed, which might improve the prognosis of this neoplasm that remains dismal based on conventional chemotherapy.

**Abstract:**

Peripheral T-cell lymphoma, not otherwise specified (PTCL_NOS) corresponds to about one fourth of mature T-cell tumors, which overall represent 10–12% of all lymphoid malignancies. This category comprises all T-cell neoplasms, which do not correspond to any of the distinct entities listed in the WHO (World Health Organization) Classification of Tumours of Haematopoietic and Lymphoid Tissues. In spite of the extreme variability of morphologic features and phenotypic profiles, gene expression profiling (GEP) studies have shown a signature that is distinct from that of all remaining PTCLs. GEP has also allowed the identification of subtypes provided with prognostic relevance. Conversely to GEP, next-generation sequencing (NGS) has so far been applied to a limited number of cases, providing some hints to better understand the pathobiology of PTCL_NOS. Although several pieces of information have emerged from pathological studies, PTCL_NOS still remains a tumor with a dismal prognosis. The usage of CHOEP (cyclophosphamide, doxorubicin, vincristine, prednisone, etoposide) followed by autologous stem cell transplantation may represent the best option, by curing about 50% of the patients whom such an approach can be applied to. Many new drugs have been proposed without achieving the expected results. Thus, the optimal treatment of PTCL_NOS remains unidentified.

## 1. Introduction

Peripheral T-cell lymphomas (PTCLs) correspond to a heterogeneous group of neoplasms arising from mature, post-thymic (hence “peripheral”) T-lymphocytes [1]. They represent 10–12% of all non-Hodgkin lymphomas (NHLs) and a significant proportion of aggressive lymphomas [1]. The World Health Organization (WHO) Classification of Tumors of Haematopoietic and Lymphoid Tissues divides PTCLs into nodal, extranodal, and leukemic types, each including multiple distinct disease entities [1]. Those not fulfilling the criteria for the diagnosis of any of these entities are called PTCL not otherwise specified (PTCL_NOS). PTCL_NOS is the commonest form of T-cell tumor, which turns out to be a kind of Pandora’s box due to its extreme morphologic heterogeneity.

The category was the object of a profound revision in the Revised 4th Edition of the WHO Classification [2]. In fact, neoplasms other than angioimmunoblastic T-cell lymphoma (AITL) but showing a T-follicular helper (TFH) profile, which in the past had been included in the PTCL_NOS chapter, were moved to the new group of nodal peripheral T-cell lymphomas of TFH origin. The latter is characterized by a distinctive morphology (small-medium sized cells with clear cytoplasm), expression of at least two but preferably three TFH-associated markers (among BCL6, CD10, PD1/CD279, ICOS/CD278, SAP, CXCL13, and CCR5), gene expression profile, and mutational landscape [2,3,4].

This review focuses on the pathobiology, clinics, and therapeutic perspectives of PTCL_NOS, aiming to assist the reader in problem solving and decision making in such a complex field of haemato-oncology.

## 2. Epidemiology and Etiology

Currently, the T-cell Project (TCP) and the Comprehensive Oncology Measures for Peripheral T-cell Lymphoma Treatment (COMPLETE) registries are prospectively enrolling PTCL patients [5,6]. They provide comprehensive information on patient characteristics, clinicopathological features, prognosis, treatments, and outcomes [5,6]. Data from these registries confirm PTCL-NOS as the commonest subtype of PTCL in North America and Europe, with a frequency ranging between 22 and 36% [5,6]. In Asia, adult T-cell lymphoma/leukemia (ATLL) has the highest prevalence, at about 25%, with PTCL_NOS being second at 22%. On racial grounds, data from the population-based US Surveillance, Epidemiology, and End Results (SEER) cancer registry show a higher incidence of PTCL_NOS in Blacks compared to Hispanic and non-Hispanic whites, Asian/Pacific Islanders, American Indians, and Alaskan natives [7]. The median age at presentation is about 60 years with a male to female ratio of about 1.9:1. PTCL_NOS is exceptional in children. Reported risk factors include a history of celiac disease, psoriasis, and cigarette smoking for 40 or more years compared with non-smokers and a family history of hematologic malignancies [8]. In a small percentage of cases, neoplastic cells carry Epstein-Barr virus (EBV) infection, although EBV positivity is more commonly detected in B-cells belonging to the microenvironment [2].

## 3. Localization and Clinical Features

PTCL_NOS more frequently occurs at the nodal level, although any anatomic site can be involved [2,5,9]. Presentation in stage III–IV occurs in 85% of the cases, with secondary involvement of the bone-marrow, spleen and extranodal sites [2,5,9]. A leukemic spread is indeed rare, as is erythroderma [2,5,9,10]. The tumor can primarily present in the skin, gastro-intestinal tract (GIT), lung and central nervous system [2,5,9]. Under these circumstances, one of the distinct entities primarily occurring in the skin or GIT should be excluded. B symptoms are common, as is IPI (International Prognostic Index) ≥ 3 [2,5,9]. Eosinophilia, pruritus or hemophagocytes are variably observed [2,5,9]. Anemia (Coombs-negative) is detected in about 35% of cases, while hypergammaglobulinemia (≥16 g/dL) is recorded in less than one quarter of the patients [2,5,9].

## 4. Microscopy

**Figure 1:**
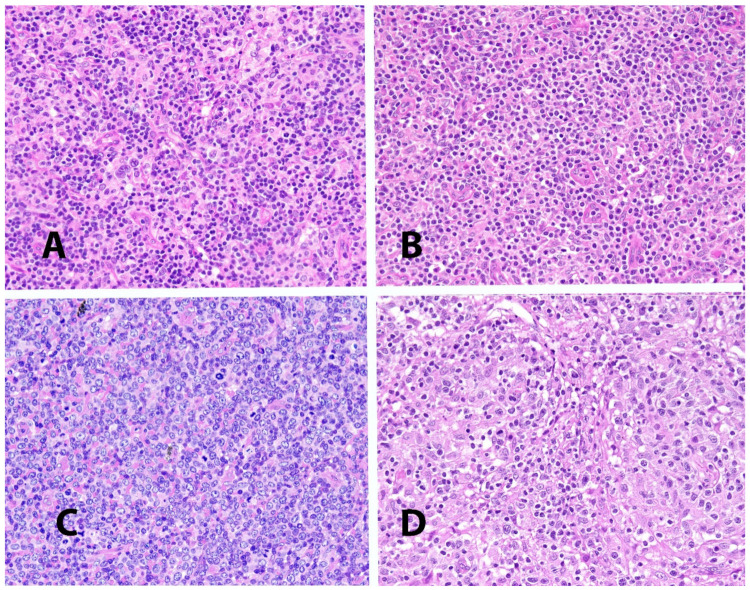
(**A**–**C**): Variability of morphologic details in different examples of PTCL_NOS. (**D**): Lympho-epithelioid variant (so-called Lennert’s lymphoma) of PTCL_NOS (all images are taken from cases stained with hematoxylin and eosin at a 40× magnification).

Cytological features are extremely variable, encompassing a spectrum ranging from a highly polymorphic to a monotonous cell population (Figure 1) [2]. More frequently, a mixture of small-medium sized elements is seen, characterized by nuclei showing irregular profiles (peanut-like, jellyfish-like, embryo-like), disperse chromatic and a distinct nucleolus, and a narrow rim of acidophilic, rarely clear, cytoplasm. In this context, a varying amount of large blasts with prominent nucleoli and a broad rim of amphophilic or basophilic cytoplasm is encountered. Less commonly, the growth consists almost exclusively of large blasts. The number of mitotic figures is usually high. B-cells, at times EBV-infected, can be present within the neoplastic population. Inflammatory components are also frequently found, consisting of eosinophils, neutrophils, histiocytes and plasma cells. 

On architectural grounds [2], if the process affects the lymph node, the normal structure is completely effaced possibly with some remnants of the B-cell zone at the periphery of the organ. Conversely to what observed in AITL, there are no partially open sinuses, hyperplasia of follicular dendritic cells and abundant branching endothelial venules. Infiltration of perinodal tissues can be detected. At extranodal sites, the neoplastic process produces variable alterations of the normal structure depending on the organ involved.

Lympho-epithelioid PTCL_NOS, also known as Lennert’s lymphoma, represents a peculiar variant of the tumor (Figure 1) [2,11]. It is characterized by numerous epithelioid elements, frequently giving rise to micro-granulomas, which to some extent can obscure the neoplastic population. The latter consists of monotonous small elements, with roundish nuclei, dense chromatin and a narrow rim of cytoplasm. In this context, blastic elements belonging to the neoplastic population are seen with prominent nucleoli and a rather large rim of basophilic cytoplasm. A few inflammatory elements can also be seen. The condition should be distinguished from classic Hodgkin’s lymphoma: phenotypic and molecular studies significantly contribute to the differential diagnosis.

The follicular variant of PTCL_NOS quoted in the 2008 4th edition of the WHO Classification of Tumours of Haematopoietic and Lymphoid Tissues [1] was moved to the chapter of angioimmunoblastic lymphoma (AITL) and other tumors of the TFH derivation in the 2017 revision of the Classification [2]. On this occasion, neoplasms with a diffuse growth pattern but showing a TFH profile, in the past included among PTCLs/NOS, were also reallocated in the new chapter [2].

## 5. Immunophenotype

**Figure 2:**
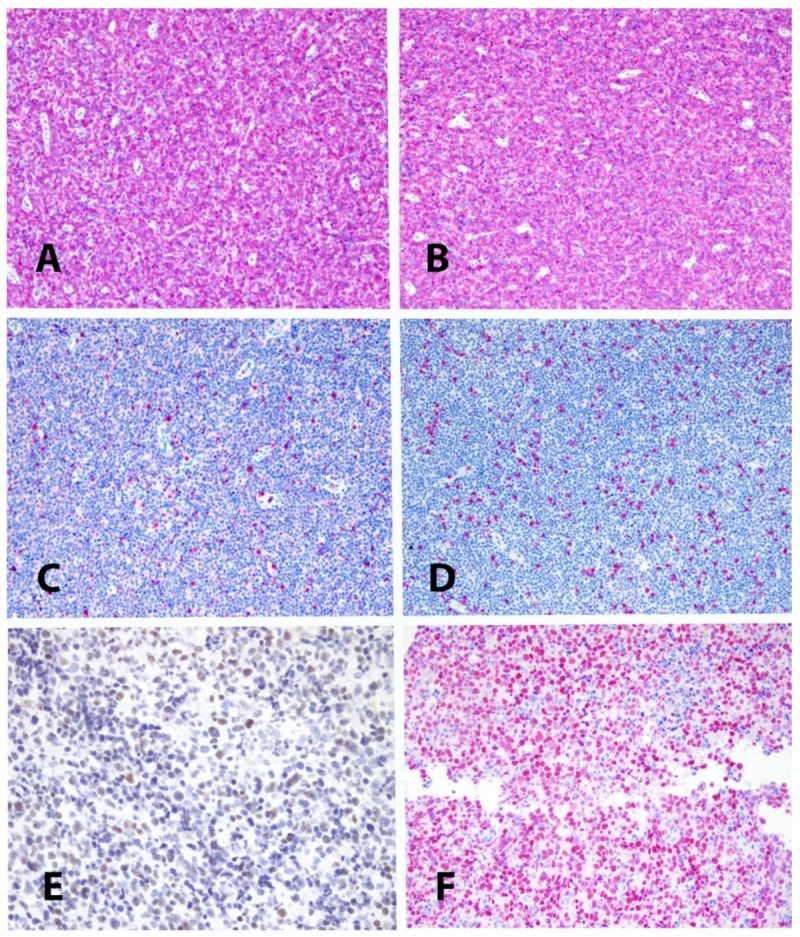
(**A**,**B**): expression of CD2 and CD3 in a PTCL_NOS; (**C**,**D**): defectivity of CD5 and CD7 in the same case (Immunoalkaline-phosphatase, Gill’s hematoxylin nuclear counterstain, 10×). (**E**): example of expression of GATA3 in a PTCL_NOS (immunoperoxidase, Gill’s hematoxylin nuclear counterstain, 20×). (**F**): example of expression of TBX21 in a different PTCL_NOS (Immunoalkaline-phosphatase, Gill’s hematoxylin nuclear counterstain, 20×).

One of the main features of PTCL_NOS is the loss of one or more of the T-cell associated antigens, more frequently CD5 and CD7 [12] (Figure 2). Interestingly, CD4 and CD8 can be both defective or co-expressed in about 40% of cases [12]. The global profile of T-cell associated antigens allows the distinction between neoplastic and reactive T-cells (defective vs. complete). In more than 50% of the cases, there is CD30 positivity in a proportion of neoplastic cells ranging from 25 to about 100% [13,14]. In the latter case, an ALK-negative anaplastic large cell lymphoma (ALK^-^·ALCL) should be excluded, a fact that is based on cell morphology and immunohistochemistry (ALK^-^·ALCL being usually characterized by a null profile, EMA positivity and cytotoxic phenotype). CD30 positivity is of interest in light of the therapeutic usage of Brentuximab-Vedotin. In this respect, it has been reported that a cut-off value of 10% suffices to have a response to the conjugated antibody [15]. The rate of positivity for the nuclear associated antigen Ki-67 is usually high, varying from 30 to more than 90% of the neoplastic cells [12]. Most cases turn out to be positive at the staining for βF1, while a few express γ/δ phenotype or are T-cell receptor (TCR) double-negative [16]. BCL2 is expressed in most if not all cases. Expression of CD38 and CD52 is variably recorded. In particular, CD52 is detected in about 40% of cases, in keeping with the results of gene expression profiling, which shows downregulation of the homologous gene [17]. The latter finding is provided with clinical implications (see below).

The search for the TFH-related markers BCL6, CD10, PD1/CD279, ICOS/CD278, SAP, CXCL13, and CCR5 should be routinely performed to allow the distinction from other nodal PTCLs of TFH origin, which express at least two of them and were in the past included in the PTCL_NOS chapter [2,3,4]. The positivity for only one of these molecules still justifies the diagnosis of PTCL_NOS. For instance, PD1/CD279 is not an absolute marker of TFH cells, as often thought. In fact, it is expressed by exhausted T-lymphocytes irrespective of the subset they belong to [18].

Additional markers useful for an accurate diagnosis are TBX21, GATA3, CXCR3, and CCR4 [19]. They allow one to surrogate the molecular subclassification produced by GEP [20]. In particular, the cases co-expressing TBX21 and CXCR3 are provided with a more favorable course than those simultaneously positive for GATA3 and CCR4 or negative for all these markers (Figure 2). The group of TBX21-related tumors also includes cytotoxic cases, expressing TIA-1, Granzyme B and/or perforin. Their identification is indeed important, since they are provided with the worst prognosis among PTCLs/NOS, with the exception pf Lennert’s lymphoma. The latter, which is characterized by a cytotoxic non-activated phenotype (TIA1+, Granzyme B−, and perforin), has been reported to have a more favorable clinical course [9].

CD56 and CD20 are occasionally and aberrantly expressed [2,21]. The cases expressing CD20 turn negative at the determination of other B-cell markers, including CD19, CD79a and PAX5. The ones CD56-positive should be differentiated from extranodal NK/T-cell lymphoma, nasal type and monomorphic epitheliotropic intestinal T-cell lymphoma. The former is characterized by the distinct clinical presentation, angiocentricity, extensive necrosis, usual intracytoplasmic expression of CD3 epsilon, regular positivity for cytotoxic markers and EBV, and frequent detection of the megakaryocyte-associated tyrosine-kinase (MAKT) [2]. The latter shows characteristic localization at the intestinal level (especially small intestine), monotonous morphology, usual lack of CD5, expression of CD8, positivity for TIA, and possible detection of MATK [2].

Finally, as mentioned above, a few PTCLs/NOS reveal EBV infection by in situ hybridization with EBER1/2 probes, more often affecting scattered reactive B-cells [2,22].

## 6. Molecular Characteristics

### 6.1. T-Cell Receptor Gene (TCR) Rearrangement

The usage of the BIOMED2 approach allows the identification of a clonal rearrangement of TCR β and/or γ also by using DNA fragments extracted from formalin-fixed, paraffin-embedded (FFPE) samples [23]. Occasionally, a negative result is observed: this can occur in cases of poor fixation or early neoplastic involvement, which turns out to be below the threshold of sensitivity of a conventional Sanger approach. Under the latter circumstances, a next-generation sequencing technique for TCR or recurrently mutated genes (see below) may provide evidence of the neoplastic nature of the process [24].

### 6.2. Gene Expression Profiling

The gene expression profile (GEP) of PTCLs was the object of a series of studies in the first years of this century.

In 2004, Martinez-Delgado et al. published a report based on a DNA micro-array, which included 6386 cancer-related genes and was applied to fresh/frozen (FF) material corresponding to 5 T-lymphoblastic lymphomas (T-LbLs), 34 PTCLs, including 19 PTCLs/NOS, three cell lines derived from T-LbL, magnetically isolated T-lymphocytes obtained from a pooled peripheral blood of five anonymous donors, five reactive lympho nodes, and two normal thymuses [25]. The aim was to compare the gene signature of PTCL with the ones of T-LBL and normal T-lymphocytes. Significant differences were observed between PTCLs and T-LbLs, which included the activation of the NF-kB signaling pathway in the former. Differences were also observed between PTCLs and normal T-lymphocyte and reactive lymph nodes, corresponding to genes regulating the immune response and survival. 

In 2007, Piccaluga et al. PTCLs applied the Human Genome U133 2.0 Plus microarray to FF material from 28 PTCLs/NOS, six angioimmunoblastic T-cell lymphomas (AITLs), six anaplastic large cell lymphomas (ALCLs), and 20 samples of purified B- and T-cells, respectively [26]. They demonstrated that PTCL_NOS carried a GEP clearly distinct from that of normal T-cells. Comparison with the profiles of purified T-cell subpopulations (CD4+, CD8+, resting (HLA-DR−) and activated (HLA-DR+)) revealed that PTCLs/NOS were closer to activated peripheral T-lymphocytes, either CD4+ or CD8+. When compared with normal T-cells, PTCLs/NOS displayed deregulation of functional programs often involved in tumorigenesis (e.g., apoptosis, proliferation, cell adhesion, and matrix remodeling). Products of deregulated genes could be detected in PTCLs/NOS by immunohistochemistry with an ectopic, para-physiologic, or stromal location. PTCLs/NOS aberrantly expressed PDGFRα. Notably, both phosphorylation of PDGFRα and sensitivity of cultured PTCL cells to imatinib were found, the latter observation being of potential therapeutic relevance. The latter observation allowed the successful usage of Imatinib in a few patients with PTCL, either of the NOS or the ALCL type [27,28]. Unfortunately, this therapeutic approach has remained anecdotal without a subsequent validation study.

In 2014, Iqbal et al. published the largest series of PTCLs so far profiled from FF tissue (total number 372) [20]. They showed that the 121 cases of the NOS type had a gene signature that differed from the one of all the remining T/NK -cell tumors. Furthermore, in the setting of PTCLs/NOS, the authors reported the occurrence of three subtypes, respectively, provided with TBX21-related, GATA3-ralated and TBX21/GATA3 double-negative signatures. The first group was thought to stem from Th2 elements, while the GATA3-related one was thought to stem from Th1 cells. The distinction was provided with prognostic relevance; in fact, the TBX1-related group had a significantly better response to chemotherapy than the GAT3-related one, with the double-negative cases lying in between. Importantly, the TBX21 group included cases with cytotoxic profiles, which have the worst prognosis. Thus, its prognosis becomes even more favorable by subtracting the cytotoxic cases. At the current time, attempts are being made to transfer these signatures to a customized digital GEP. In the meantime, an immunohistochemical algorithm was developed that surrogates the results of GEP, based on the usage of four antibodies, respectively, raised to TBX21, GATA3, CHCR3 and CCR4 (see above).

In 2013, Piccaluga et al. had published a rather similar study, in which 244 PTCLs had been profiled (158 NOS, 63 AITLs, and 23 ALK-negative ALCLs) by extracting DNA from FFPE tissue samples and adopting the Whole Genome DASL technology, which, however, is no longer available, thus hampering its further application [29]. The authors identified molecular signatures (molecular classifier (MC)) discriminating either AITL and ALK-negative ALCL from PTCL_NOS in a training set, the results being further validated in an independent series of cases with DNA extracted from both FFPE and FF tissue. The overall accuracy of the MC was remarkable: 98 to 77% for AITL and 98 to 93% for ALK-negative ALCL in test and validation sets of patient cases, respectively. Furthermore, the MC significantly improved the prognostic stratification of patients with PTCL. Particularly, it enhanced the distinction of ALK-negative ALCL from PTCL_NOS, especially from some CD30-positive PTCLs/NOS with uncertain morphology. Finally, MC identified some cases originally classified as PTCL_NOS but sharing a T follicular helper (TFH) derivation with AITL, a finding that led to the definition of the new category of nodal PTCL of TFH origin of the 2017 edition of the WHO Classification.

Finally, by targeted gene expression profiling on the NanoString platform, Sugio et al. analyzed the DNA extracted from 68 PTCLs/NOS and validated their findings by immunofluorescence in tumor sections [30]. The authors, focusing on the microenvironment, showed that signatures representing tumor-infiltrating immune cells significantly influenced patients’ clinical outcomes, while those of lymphomatous elements did not. Cases carrying both B-cell and dendritic cell (DC) signatures (BD subgroup) were characterized by a favorable clinical outcome, while those lacking B-cell and/or DC signatures (non-BD subgroup) had an extremely poor prognosis. About 50% of the non-BD cases exhibited a macrophage signature. In these cases, immunofluorescence on routine sections revealed a significant macrophage infiltration. Tumor-infiltrating macrophages expressed high levels of the immune-checkpoint molecules programmed death ligand 1/2 (PDL1/2) and indoleamine 2,3-dioxygenase 1, suggesting that checkpoint inhibitors might represent a therapeutic option for patients in this subgroup.

### 6.3. Next-Generation Sequencing (NGS)

NGS has so far found limited application to PTCL_NOS. A few studies, based on targeted NGS, have revealed recurrent mutations of genes involved in epigenetic regulation (*MLL2*, *TET2*, *KDM6A*, *ARID1B*, *DNMT3A*, *MLL*, *TET1*, *ARID2*), cell signaling (*TNFAIP3*, *APC*, *CHD8*, *ZAP70*, *NF1*, *TNFRSF14*, *TRAF3*) and tumor suppression (*TP53*, *FOXO1*, *BCORL1*, *ATM*) [31,32].

A more recent study combining targeted NGS, copy number alterations (CNAs) and GEP has allowed the distinction of molecular subgroups based on the genetic drivers of oncogenic pathways [33]. Importantly, different alterations were recorded in the TBX21 and GATA3 subgroups, further underlining that the gene signature corresponds to different biological scenarios. In particular, PTCL-GATA3 exhibited higher genomic complexity characterized by frequent loss or mutation of tumor suppressor genes targeting the CDKN2A/B-TP53 axis and PTEN-PI3K pathways along with gains/amplifications of *STAT3* and *MYC*. Several CNAs, in particular loss of *CDKN2A*, exhibited prognostic significance in PTCL-NOS as a single entity and in the PTCL-GATA3 subgroup. The PTCL-TBX21 subgroup had fewer CNAs, primarily targeting cytotoxic effector genes, and was enriched in mutations of genes regulating DNA methylation. CNAs affecting metabolic processes regulating RNA/protein degradation and TCR signaling were common in both subgroups.

Rather similar results were obtained by Maura et al., by performing whole genome sequencing of five FFPE PTCL_NOS samples [34]. The authors found a high prevalence of structural variants and complex events, such as chromothripsis, likely responsible for the observed CNAs. *CDKN2A* and *PTEN* deletions emerged as the most frequent aberrations. They appeared specifically associated with PTCL_NOS, being rare and never co-occurring in AITL and ALCLs. *CDKN2A* deletion turned out to correlate with shorter overall survival in a multivariate analysis corrected by age, IPI, transplant eligibility and GATA3 expression.

By using integrated whole exome sequencing (WES), targeted capture sequencing, gene expression profiling, and immunohistochemistry, Watatani et al. also stressed the relevance of *CDKN2A* and/or *TP53* alterations in non-TFH PTCLs/NOS [35]. In particular, these alterations occurred in a subtype of PTCL_NOS, which was provided with extensive genetic instability and more aggressive clinical course. 

Two studies based on RNA sequencing (RNAseq) reported alterations of the *VAV1* gene in about 15% of PTCLs/NOS [36,37]. These alterations consist of either mutations at intron 25 or fusions causing the replacement of the C-terminal SH3 domain of *VAV1* by the calycin-like domain of *THAP4*, the SH3 domain of *MYO1F* or the EF domains of *S100A7*. All these alterations cause activation of *VAV1*, which is silent in normal T-lymphocytes, thus suggesting that the above-mentioned alterations play a major role in the process of lymphomagenesis.

Finally, Laginestra et al. performed whole-exome sequencing (WES)—integrated by RNAseq—in 21 PTCLs/NOS with FF material available [38]. Based on WES results, a panel of 137 genes was designed, which was applied to 71 additional tumors by a targeted deep approach. In addition to mutations of *TET2*, *DNMT3A*, *KMT2D*, *KMT2C*, *SETD2*, *NOTCH1, STAT3* and *NOTCH2*, observed across PTCLs, recurrent mutations of the *FAT1* tumor suppressor gene were for the first time recorded in 39% of cases. Mutations of the tumor suppressor genes *LATS1*, *STK3*, *ATM*, *TP53*, and *TP63* were also observed, although at a lower frequency. Conversely, no mutations were found in *RHOA*, *IDH2*, and *CD28*, known to occur in AITL and other nodal T-cell lymphomas of TFH origin. In addition to coding for the homologous protein, which belongs to the cadherin superfamily, *FAT1* assembles a multimeric Hippo signaling complex, resulting in the activation of core Hippo kinases. In particular, somatic mutations in *FAT1* and Hippo core molecules might represent an important pathogenetic event in a percentage of PTCLs/NOS. They turned out to be independent of the GATA3 and TBX21-related subgroups and associated with a distinctive signature, significantly enriched in genes involved in cell growth and migration, apoptosis and invasiveness. Notably, patients with *FAT1* mutations showed inferior overall survival compared to those with wild-type *FAT1*.

## 7. Prognosis and Therapy

### 7.1. Prognostic Indexes

Several clinical prognosticators have been proposed in recent years. The International Prognostic Index (IPI) does represent a tool transversally applied to all non-Hodgkin lymphomas but not specifically developed for PTCL_NOS [39]. Conversely, four prognostic indicators have been reported expressively pointing to PTCL_NOS. The Prognostic Index for peripheral T-cell lymphoma, unspecified (PIT) was based on age (<60 years), ECOG PS ≥ 2, LDH level upper normal range, and bone-marrow involvement [40]. According to the results of an Italian cooperative study, the outcome of PTCLs/NOS significantly varied depending on the presence of one, two or three or more of the PIT factors. In this setting, the substitution of bone-marrow involvement with Ki-67 marking ≥80% resulted in a more robust tool (modified-PIT) [12]. A further score (IPTCLP) was proposed based on the data gathered in the course of the International T-cell Project Network and including serum albumin, performance status, stage and absolute neutrophil count [41]. Finally, the International Peripheral T cell Lymphoma Project developed an index based on the presence of B-symptoms, bulky disease ≥ 10 cm, elevated serum C-reactive protein, a high number of transformed tumor cells, and platelet count less than 150 × 10^9^/L, which adversely affected both overall (OS) and progression-free survival (PFS) in univariate analysis, although only bulky disease remained predictive for both OS and PFS, and thrombocytopenia for PFS in multivariate analysis [9]. In this study, the PIT and IPI remained highly significant for both OS and PFS [9].

### 7.2. Chemotherapy

PTCL_NOS has a dismal prognosis, its cure representing an unmet need. In fact, this lymphoma subtype shows low sensitivity to anthracycline-based regimens successfully applied to B-cell neoplasms (e.g., CHOP (cyclophosphamide, doxorubicin, vincristine, prednisone)). Only 40% of patients are alive at 36 months by adopting standard therapies [40]. In the case of relapse, the prognosis is even worse, with an overall survival probability of about 20% at 3 years [41]. A major limitation of the current knowledge in the setting of PTCL_NOS therapy is due to the fact that management strategies of PTCL_NOS have been derived from the data of several studies composed by mixed PTCL subsets [41,42,43,44]. The most widely used approach at the time being was proposed by d’Amore et al. in 2012 [45], consisting of the usage of induction CHOEP regimen (cyclophosphamide, doxorubicin, vincristine, prednisone, etoposide) followed by autologous stem-cell transplantation (ASCT) as first line consolidation. Current PTCL treatment guidelines recommend the addition of etoposide to standard CHOP (CHOEP regimen) as first line therapy in young (<60 years of age) fit patients [46]. This recommendation is based on data from a retrospective study on 289 patients with PTCL treated with 6–8 cycles of standard CHOP or CHOP plus etoposide, demonstrating an event free survival (EFS) advantage for the CHOEP arm in patients with less 60 years of age with normal lactate dehydrogenase (LDH) levels. Notably, in this study, the vast majority of patients had ALCL (n = 191), and only 24% (n = 70) of the whole cohort were designated as PTCL-NOS [47]. A second retrospective study demonstrated a similar progression-free survival (PFS) advantage for young (<60 y) patients treated with CHOEP [48]. In the latter study, 34% of patients had a PTCL-NOS diagnosis [48]. From the clinical point of view, data from retrospective studies are often difficult to interpret and the PTCL field is particularly prone to interpretation biases, given the heterogeneity of PTCL subtypes considered in clinical studies, which often complicate the generalization of the results, limiting their applicability in clinical practice. In any case, the addition of etoposide to CHOP did not dramatically change the poor outcome of PTCL_NOS; therefore, alternative treatment backbones are urgently needed.

### 7.3. First Line Intensification: Autologous and Allogeneic Stem Cell Transplantation

In the original study from D’Amore and colleagues [45], analyzing a rather heterogeneous cohort comprising different PTCL histologies, about 50% of patients receiving this treatment were alive at five years. Despite its widespread use, the role of first line ASCT consolidation in the PTCL_NOS is still debated. In fact, the value of this strategy has been addressed in a subsequent study by Park and coworkers [49], showing that whether ASCT in first complete remission (CR) could be of value in high-risk PTCL patients as a whole, it did not provide a significant PFS and OS advantage when the analysis was restricted in the specific PTCL_NOS subset. The role of first line allogeneic stem transplant (allo-SCT) consolidation has been evaluated in retrospective and prospective studies [43,50]. A recent phase 3 randomized study from Schmitz and coworkers [50] investigated the role of allo-SCT consolidation in first CR in PTCL. While confirming a strong graft versus lymphoma (GVL) effect and thus a lower relapse rate, this study did not demonstrate a significant OS advantage for allo-SCT, due to increased transplant related mortality (TRM) compared to ASCT. Allogeneic SCT (allo-SCT) is a valuable therapeutic option for patients with relapsed/refractory disease [51]. The identification of the most appropriate timing, conditioning regimen, and donor type continues to be the object of research [52]. Furthermore, clinically applicable tools for the identification of specific patients with high-risk disease who could benefit from allo-SCT in first remission need to be further elucidated. However, a common message from most studies evaluating the efficacy of first line treatment strategies is that a substantial fraction of patients do not achieve CR with regimens based on the CHOP-backbone, refractoriness to first line treatment being the main adverse prognostic factor for survival.

### 7.4. Novel Therapies

These observations and the fact that treatment intensification cannot be applied to elderly patients due to toxicity imply that the identification of novel more effective front line therapies is a key unmet need that should be prioritized. In the past few years, new targeted agents have generally provided inferior results as compared to the ones achieved in B-cell lymphoma, probably due to the lack of effective and systematic biomarker discovery studies. Histone deacetylase inhibitors (HDACi) have demonstrated remarkable benefits in at least 20–25% patients with PTCL_NOS [53], which is in line with preclinical studies showing genomic alterations in epigenetic modulators in a similar fraction of cases. On the contrary, HDACi show higher activity in AITL, where genomic alterations of epigenetic modulators play a major pathogenic role [53]. In addition, HDACi efficacy does not seem to change as a function of prior therapies, thus suggesting some predisposed vulnerability that does not share the same cross-resistance mechanisms with conventional chemotherapy. These and other observations suggest that HDAC inhibitors could synergize with a host of drugs active in PTCL_NOS and therefore could play a more significant role in combination therapies. In this light, although preliminary data from studies investigating combinations of HDACi and conventional chemotherapy provided promising results [54], recent findings do not support the addition of HDACi to conventional CHOP chemotherapy [55]. The anti-CD30 monoclonal antibody SGN-30 conjugated with monomethyl auristatin E Brentuximab-Vedotin (BV) represents another interesting tool for the treatment of CD30+ PTCL. In the ECHELON-2 trial, the addition of BV to CHP (CHOP regimen without vincristine) provided significant PFS and OS advantage in combination with first line chemotherapy. However, since 75% of enrolled patients had a diagnosis of ALCL (which is ubiquitously CD30+), and the study was not powered enough to demonstrate a PFS advantage for individual PTCL subtypes, these results can be considered practice changing only for ALCL [56]. In fact, in line with the varying and inconsistent expression of the CD30 molecule in PTCL_NOS cells, data from the ECHELON-2 trial cannot be extrapolated and generalized to all PTCL subtypes without the risk of important interpretation biases. For these reasons, the European Medicines Agency (EMA) approved BV in combination with CHP only for the treatment of newly diagnosed ALCL. As mentioned before, the cut-off value of CD30-positive neoplastic elements is still matter of debate. Preclinical evidence suggests a possible role of PI3K inhibitors in GATA-3 PTCL_NOS, which should be confirmed in future clinical studies [57]. Other potential therapeutic targets are BCL2 [58,59], CD38 [59], CD52 [17], and PDGFRα/β [27,28,60]. In particular, while the expression of PDGFRα/β might represent the rationale for the usage of tyrosine-kinase inhibitors [27,28], the other molecules can be targeted by specific small molecule inhibitors and monoclonal antibodies (i.e., Venetoclax, Daratumumab, and Campath-1). The anti CD52 Campath-1 antibody has provided conflicting results, given the high rates of infections due to the profound immune suppression induced by CD52 targeting, which limits its efficacy. In line with this, a recent trial of Campath-1 in combination with CHOP chemotherapy could not demonstrate a PFS and OS advantage for the experimental arm, despite higher response rates [61,62]. In general, the poor efficacy of standard CHOP chemotherapy in the PTCL_NOS subset challenges the exploitation of CHOP as a backbone for novel combinations with targeted agents. Novel promising chemo-free backbones are under evaluation at the time being, including HDACi-based combinations and PI3Ki-based regimens. For example, the association of Romidepsin with 5-Azacytidine was shown to have high efficacy in a recently published phase 2 trial [63] and the PI3K inhibitor Duvelisib produced high response rates as single agent or in combination [64]. Finally, novel immunotherapeutic approaches are currently under investigation in PTCL. Regarding immune checkpoints inhibitors, phase 1 data showed promising efficacy in a small cohort of PTCL, providing the rationale for further clinical investigation [65]. Early reports produced concerns about lack of efficacy and possible detrimental effects of anti PD-1 therapy in PTCL, such as hyper progression [66,67]; however, the relative number of PTCL_NOS patients enrolled in these trials was too small to draw any definite conclusion for this specific PTCL subtype. A more recent trial investigating the efficacy and safety of the anti PD1 antibody geptanolimab in a larger PTCL cohort showed a 17.9% overall response rate (ORR) for the PTCL_NOS subset [68]. Of note, in this trial the highest ORR was observed in the NK/T-cell lymphoma subset in line with earlier reports [69]. The specific role of other forms of immunotherapy such as bispecific antibodies and CAR-T cell therapies cannot be addressed at the present time due to insufficient data regarding the PTCL_NOS subtype.

## 8. Conclusions

Since 2000, knowledge on the pathobiology of PTCL_NOS has been progressively expanding thanks to the application of an array of phenotypic and molecular techniques. The latter have allowed the identification of subtypes, provided with prognostic relevance, as well as of potential therapeutic implications, which have led the proposal of novel regimens. Mature data on the efficacy of these novel regimens are, however, still missing, also due to the rareness of PTCL_NOS. In the future, dedicated collaborative studies should address the value of novel treatment strategies according to the PTCL subtypes, given the observed differences in mutational landscapes and response to new drugs. The hope is to find innovative approaches which can definitively improve the PTLC_NOS clinical course and prognosis.

## Data Availability

Not applicable.
